# Decolonial knowledge in Practice: a mestiza reflection on sentipensar in indigenous Nasa epistemologies

**DOI:** 10.3389/fsoc.2025.1605618

**Published:** 2025-09-25

**Authors:** Paola Chaves Pérez

**Affiliations:** Nijmegen School of Management, Radboud University Nijmegen, Nijmegen, Netherlands

**Keywords:** decolonial knowledge, indigenous knowledge, embodiment/bodily experience, political epistemology, coloniality of knowledge, epistemic injustice, critical research methods, Latin American decolonial thought

## Abstract

This paper presents the concept of sentipensar in the Nasa Indigenous practices. Through the concept of sentipensar this article shows that Nasa knowledge can emerge from being within relationships through the body, through affect, through intuition, and through deliberation processes that listen the body and the spirits. It affirms that knowledge is not only produced in the mind, but also in rituals, in land-based practices, and in the everyday acts of care and resistance that sustain community life. As such, *sentipensar* offers an understanding of knowledge that is deeply situated and communal, challenging the dominant view of knowledge as a cognitive or individual pursuit.

*Celebration of the Marriage of Heart and Mind by Eduardo Galeano*

*Why does one write, if not to put one's pieces together? From the moment we enter school or church, education chops us into pieces: it teaches us to divorce soul from body and mind from heart. The fishermen of the Colombian coast must be learned doctors of ethics and morality, for they invented the word sentipensante, feeling-thinking, to define language that speaks the truth (Galeano, 2004, p. 89)*.

## On epistemic dualisms and sentipensar

For several years, disciplines such as sociology, philosophy, and anthropology have seriously questioned their Eurocentrism and colonial practices in knowledge production. The result has been the development of new fields of work in the social sciences—such as postcolonial theory, subaltern studies, the decolonial turn, posthumanities, and feminist epistemologies—that have changed the perspective from which classical disciplines are practised, affecting their methodologies, mainly qualitative research methods. While conventional methodologies—especially within positivist social sciences—tend to reinforce epistemic dualisms, postcolonial, decolonial, and feminist perspectives have long questioned these divides. These dualisms shape not only what counts as knowledge, but also how researchers relate to the people they work with, often producing extractive dynamics in the name of objectivity or expertise (Pillow, 2003). For instance, the separation between the knowing subject and the external object of knowledge produces a dehumanisation of racialised bodies in academia (Maldonado-Torres, 2007) and turns knowledge production into a hierarchical and colonial relation of domination (Quijano, 2000; Mignolo and Escobar, 2010); the separation between knowledge and culture reinforces an inherent classification whereby knowledge is generated in what we call today the Global North or through Global North perspectives, while the Global South has cultures (Blaser, 2013; Law, 2015); moreover, academics produce science, whereas Indigenous peoples possess wisdom (Mignolo, 2009); the separation between humans and nature, promotes an anthropocentric worldview suggesting that humans are the centre and most significant entities in the world (Viveiros de Castro, 2014; De La Cadena, 2015; Escobar, 2018b); finally, the separation between rationality and emotion suggests that rationality is objective and neutral, whilst emotion is subjective and biassed. Hence, reliable knowledge can only be achieved through methods that eliminate the personal values and emotions of individual scientists (Haraway, 1988; Jaggar, 1989). These dualisms struggle to recognise inter-subjectivity, reciprocity, and the ethical dimensions of research relationships (Pillow, 2003; Smith, 2008; Walsh, 2015). Moreover, these assumptions undermine knowledge systems that incorporate emotion, spirituality, or relationality—often those from Indigenous, African, or decolonial feminist traditions (Lugones, 2010).

Feminist epistemologists such as Harding (2008) and Haraway (1988) have long critiqued the presumed objectivity and neutrality of scientific knowledge, highlighting instead its situated, embodied, and gendered character. Sara Ahmed (Ahmed and Stacey, 2001; Ahmed, 2014) expands this perspective by showing how emotions are not private states but circulate through bodies and collectives, shaping orientations and defining what is felt as familiar, threatening, or worth knowing. Her work helps reframe feeling as central to knowledge, not it's opposite. Similarly, Braidotti (2019), a feminist philosopher working within the posthumanist tradition, foregrounds affect and relationality in her critique of disembodied, rationalist knowledge regimes. Her work contributes to dismantling the figure of the disembodied, purely rational subject, opening space for alternative ways of knowing that are situated, emotional, and interconnected. Even within moral philosophy, Nussbaum (2004) argues that emotions are not opposed to reason, but are themselves forms of evaluative judgment—ways of discerning what matters, demonstrating how they are deeply intertwined with ethics, reasoning, and human development. These perspectives converge with neuroscience and embodied cognition theories (e.g., Damasio, 1999), which affirm that emotion is essential to decision-making and thought. In methods, sensory ethnography, in particular, offers tools to engage with lived experience through multisensory attention, helping researchers stay close to how people perceive and make sense of the world through their bodies (Pink, 2015).

These perspectives have opened important epistemological spaces that go beyond reductive, mechanistic models of science. Yet, despite these advances, much of institutionalised knowledge production remains bound to disciplinary silos and entrenched dualisms. Academic institutions in Western and Westernised societies, continue to operate largely through these epistemic dualisms, assuming a “zero-point epistemology” that favours colonial, Western, upper-class and paternalistic, and methodological perspectives detached from embodied knowledge (Mignolo, 2009; Ramirez, 2021). Thus, not only does the location of knowledge production remain invisible (context), presenting itself as neutral, but also the bodies that produce it are rendered invisible, reinforcing epistemic homogenisation (Mignolo, 2009). Moreover, this favours white, Western masculine bodies holding an invisible and privileged position, unmarked by their bodily characteristics (Braidotti, 2019; Ramirez, 2021).

When living in Latin America, it is evident that coloniality continues to influence the production of knowledge today. Not only does it position science and Latin American research in a state of peripherality and dependence, but it also fosters a selective deafness within the region's own research. Aníbal Quijano explains that in the order established by coloniality, which persists in contemporary knowledge practices, colonised peoples were not only politically and economically dominated but also intellectually disempowered (Quijano, 2000). Their knowledges were extracted, erased, or transformed to fit Western paradigms (Quijano, 2000). To reverse this, the decolonial turn has challenged the idea of one universe with multiple interpretations by placing Indigenous knowledge as ontologies or universes of knowledge (Castro-Gómez and Grosfoguel, 2007; Law, 2015). The idea is to acknowledge that Indigenous people possess not mere pieces of knowledge, but rather clear systems for understanding and studying the world. To express this, the term “pluriverse” has been coined, referring to a world in which many worlds are possible (Escobar, 2017; Blaser and de la Cadena, 2018; Bastidas Aguilar, 2020).

In contrast to epistemic dualisms, Indigenous ontologies are relational and, rather than understanding humans, nature, subjects, and objects separately and conceptualising them as isolated units with fixed properties that interact with one another, Indigenous ontologies view different elements as constituted and defined by their relationships with other elements (Castro-Gómez and Grosfoguel, 2007; Mignolo and Escobar, 2010; Ingold, 2011; Viveiros de Castro, 2014; De La Cadena, 2015; Escobar, 2018a). Several scholars have pointed to the deeper roots and political urgency of relational ontologies in Indigenous thought. For instance, Indigenous knowledge of ecology has become a vital component of various approaches to sustainable development, embraced by different researchers and political groups (Fernando, 2003). The environmental crisis has recognised the critical role of Indigenous practices in preserving ecosystems and ecosystem governance (Agrawal, 1995; Popova, 2014; Tengö et al., 2014). For instance, Tengö et al. (2014) explain that diverse knowledge systems are necessary to enhance ecosystem governance, in which indigenous knowledge plays a key role. However, many authors like Todd (2016) and Escobar (2018a) caution against the appropriation of relationality from Indigenous cosmologies without recognising their ontological and epistemological distinctiveness. Similarly, Agrawal (1995) warns about the risk of essentialising Indigenous knowledge and reducing it through integration into dominant systems. Furthermore, Fernando (2003) shows that the decontextualisation and “scientification” of Indigenous knowledge by some NGOs can result in epistemic violence, as it strips knowledge from its lived, communal, and spiritual foundations. Scientification here refers to the “validation” of indigenous knowledge through the scientific method.

A relational and contextualised approach to indigenous knowledge is a crucial aspect of the discussion in this article. Therefore, I focus on two interconnected themes that run through this article. One concerns the relationship between the researcher and the communities, which I explore through reflections on my positionality and fieldwork experiences. The other engages with the interplay between rationality and emotion, as I observed it emerging in the practices and everyday life of Nasa communities. I use the concept of *sentipensar* as a conceptual tool, which recognises the importance of the connection between feelings and thinking as part of decision-making processes. *Sentipensar* is a common practice among various Indigenous, farmer, and fishermen groups across Colombia. It was coined in the 1970s by Colombian sociologist Orlando Fals Borda, stemming from his extensive experience conducting Participatory Action Research (PAR) in the rural areas of Colombia (Fals Borda, 2015). *Sentipensar* expresses the inseparability of reason, emotion, body, spirit, and land. It is rooted in local and collective knowledge traditions as a relational and dialogical way of knowing, cultivated through shared reflection and action in the territories (Fals Borda, 2015). It refers to thinking with the body or heart and implies to think with feeling and to feel with thinking, simultaneously. Indeed, the whole point of *sentipensar* is to erase the Cartesian divide between thinking and feeling, and to propose that to think, one must also feel.

*Sentipensar* avoids the hierarchisation in which rational thinking is superior to our feelings, in which feelings must always be controlled or supervised by reason. It resists the scientific ideal of neutrality, proposing instead a form of engaged inquiry that values affect, territory, and culture (Fals Borda, 2015; Rodriguez Castro, 2018). For Fals Borda, this mode of knowing is not only epistemological but also political: it underpins a practice of liberation that connects popular wisdom, participatory research, and social transformation (Fals Borda, 2015). The Zapatistas in Chiapas call it, *co-razonar*, and according to Escobar (2014) it refers to how territorialised communities have a profound integration of thought and emotion. Hence, *sentipensar* refers to the embodied experience of knowledge production in one specific context (Rodriguez Castro, 2018). In this way, *sentipensar* urges us to engage empathetically with the territories, cultures, and knowledge systems of indigenous communities, embracing their ontologies while challenging the scientific dependence on decontextualised knowledge that forms the basis of conventional notions of “development,” “growth,” and “economy” (Escobar, 2014). Nowadays, *sentipensar* has gained traction among Latin American scholars who draw on decolonial and feminist perspectives, and it is increasingly used to reflect on the embodied and relational dimensions of fieldwork, shaped by territory and territorial practices (Rodriguez Castro, 2018).

The concept of *sentipensar* did not come to me in a straightforward way. I had read about it during my bachelor, and it resonated quickly, but it was during my PhD that I understood it. The PhD fieldwork was a deeply personal and confrontational experience, during which I felt I lacked the cognitive tools to make decisions and understand the environment surrounding me. I was trying to use my reason to make decisions in a very uncertain context. After spending a few months living in Santander de Quilichao, working with Nasa indigenous leaders and mainly joining the Indigenous Guards in their activities, I began to apply the concept of *sentipensar* in my daily life and decisions during fieldwork. This implied an understanding of my embodied experience as shaped by the specific contexts and challenges of everyday life, with direct implications for my research processes and meaning-making (Rodriguez Castro, 2018).

In this article, I explore how the concept of sentipensar, as a form of decolonial knowledge, becomes visible and meaningful through the practices and epistemologies of the Nasa people. I do so with an acute awareness that this process involves what Viveiros de Castro (2004, 2014) calls a *controlled equivocation*: recognising that translating Indigenous practices and concepts for a Western academic audience inevitably involves asymmetries and partial understandings. *Controlled equivocation* requires holding space for ontological divergence without reducing one worldview to another. From this perspective, I understand *sentipensar* as a relational and embodied way of knowing, something I learned by sharing experiences with the Nasa people, for whom the territory and the body are the primary sites of knowledge production.

This research joins a recent perspective in which *sentipensar*, a concept rooted in the epistemologies of the Global South, offers a valuable approach—both conceptually and methodologically—for enriching anthropological research Rodriguez Castro, (2018). Moreover, these reflections form part of a broader effort to engage with the decolonisation of academic knowledge production as an epistemic and political project that challenges dominant paradigms by embracing ontological pluralism and striving for both epistemic and ontological justice (Méndez Torres et al., 2013; Fúnez-Flores, 2016; Rodriguez Castro, 2018; Bastidas Aguilar, 2020; Rodríguez Castro, 2021; Ludwig et al., 2024). Yet, it is crucial to acknowledge that decoloniality is not only a matter of theoretical engagement. While scholars debate and conceptualise its implications, many farmers and Indigenous communities across the Global South are enacting decolonial practices daily—as a means of survival and resistance in their ways of living, producing, and relating to the world (Espinosa Miñoso, 2017; Espinosa Miñoso et al., 2021).

## The challenges of positionality

This article is based on my own experience using Participatory Action Research (PAR) during my PhD fieldwork, living in Santander de Quilichao and working at the headquarters of the ACIN, the indigenous Nasa Organisation in the northern Cauca region of Colombia. In this article, I adopt a reflexive approach to my experiences in fieldwork. Hence, I reflect on what I learned during my fieldwork experience while remaining critically aware of how my position as a mestiza, a woman, and educated in Western institutions influenced the various stages of my research process (Callaway, 1992 cited by Pillow, 2003). From this perspective, my reflexivity is both an exercise of self-analysis and political awareness (Callaway, 1992 cited by Pillow, 2003). Now, I cannot deny my privilege as an academic, but I will problematise it a bit. As my Brazilian friend, Juliana Lins, and I discussed over coffee, we inhabit the paradox of occupying a position that grants us access, through fluency in the dominant academic language and residency in the Global North, while simultaneously rendering our knowledge suspect or invisible within European academic discussions. This conditional access, masks deeper structures of epistemic exclusion. As migrants from the Global South, our contributions are frequently questioned unless they align with Western paradigms or with prestigious academics from the Global North, revealing how inclusion often comes at the cost of epistemic conformity or external validation, rather than genuine pluralism.

My personal history and origins are rooted in the Colombian working class. My family comes from the rural areas in the South of Colombia. I was raised in a very conservative city in the south of Colombia and educated in a Catholic religious school, while my family, although not vocally progressive, had very progressive practices. I identify as a mestiza, a mixed-race woman of Indigenous and Spanish descent. However, tracing my Indigenous heritage is rather challenging. *Mestizaje* can be understood from the concept of hybridisations, defined as sociocultural processes in which discrete structures, which existed separately, combine to generate new structures, objects, and practices (Canclini, 2003). The concept of hybridisation critiques essentialist views of identity and explains certain practices in societies characterised by great diversity (Canclini, 2003). My family practices were a type of hybridisation. However, hybridisation fails to acknowledge the contradictions inherent in the processes of colonisation and mestizaje—both violent and imposed—in Latin America. Primarily, it disregards the fact that some practices have been imposed, while others have persisted despite colonisation. Furthermore, it overlooks the issue of our limited knowledge regarding our history and how these ancestral practices originated and continue to exist. Hence, being mestizo signifies being caught between two worlds, one subjugated to the other—even when there is hybridisation.

Gloria Anzaldúa elucidates *the mestiza consciousness* characterised by the tolerance for contradiction and ambiguity and the transgression of rigid conceptual boundaries, displacing dualistic thinking (Anzaldúa, 1987). This suggests an intersectional and hybrid epistemological stance, challenging rigid academic binaries. This is not always an easy position neither is comfortable. (Rivera Cusicanqui 2018) embraces the Aymara term *ch‘ixi* (pronounced “chehe” in English or “cheje” in Spanish) as a conceptual metaphor that elucidates the contradictions in the process of *mestizaje*. The Ch'ixi metaphor refers to the types of stones that display black and white spots and that from far away could look mixed in a grey colour. Thus, it denotes juxtaposed hard and whole fragments that are interwoven in multiple ways, yet remain never completely fused or dissolved (Rivera Cusicanqui, 2018). Cusicanqui uses the term “double bind,” from Gregory Bateson, as a comparable idea because this contradiction in individual terms translates into two conflicting imperatives, neither of which can be ignored, leaving the individual facing an insoluble dilemma: either of the two demands that they want to fulfil cancels out the possibility of fulfilling the other (Rivera Cusicanqui, 2018, p. 30). In this sense, the mestizaje in Latin America is understood as the contradiction of living between worlds or even between ontologies. Recognising this double bind and embracing a creative approach to living within this contradiction is called by Cusicanqui as “*ch'ixi epistemology,”* which encourages mestizos to accept the contradiction without succumbing to collective schizophrenia (Rivera Cusicanqui, 2018, p. 30). It is from a “ch‘ixi epistemology” that I write these lines, because even when I recognised myself as a mestiza, it took me a very long time to understand this positionality fully. After all, being mestiza implied being indigenous, but also, less indigenous. Maybe one of the reasons why I wanted to work with indigenous people from my country was to get to know a part of myself that has been denied by the colonial history, but from which I could see some traces in my family practices. Maybe I was looking to embrace and awaken my *mestiza consciousness*.

## The study

The Nasa people from northern Cauca are part of the Cauca Regional Indigenous Council – CRIC, the biggest and oldest indigenous organisation in Colombia. The Nasa are known for their Guardia Indígena – GI (Indigenous Guard), a network for community protection of women, men, boys, and girls who voluntarily defend indigenous territories. The GI responds to a process of historical resignification of the ancient Nasa struggles and addresses a practical need for territorial control in indigenous lands afflicted by armed conflict (Chaves et al., 2019). In 2020, the GI received the Front Line Defenders recognition for their efforts and contributions to peacebuilding (Front Line Defenders, 2020). The guards are unarmed and use only symbolic weapons, such as a bastón (walking stick), symbolising the power bestowed upon them by their community. They also use different strategies to amass power by numbers and strengthen their collective identity (Chaves et al., 2019, 2020).

I joined the Association of Indigenous Councils from Northern Cauca (ACIN), part of the CRIC, working as a collaborator within the ACIN's headquarters in Santander de Quilichao, Cauca. In the period I spent in fieldwork, the Colombian government was negotiating the Peace Agreement with the Revolutionary Armed Forces of Colombia, FARC-EP. The northern Cauca region is one of the marginalised territories in which FARC had a strong presence, and the implementation of the agreement generated immediate changes in local dynamics. The FARC controlled parts of the drug business chain and, after FARC's demobilisation in 2017, criminal organisations fought to gain control of the territory (Álvarez Vanegas et al., 2017). Nearly 10 years after the signing of the Peace Agreement, the northern Cauca region remains one of the most dangerous areas in the country. FARC dissidents, now fragmented into different groups, have taken over the drug trade in the region and compete for control, leaving civilians—particularly Indigenous communities—caught in the crossfire (Majbub Avendaño, 2025).

The PAR methodology was developed in the 70s by a group of researchers in Latin America including Fals-Borda, as a response to decontextualised knowledge production methodologies (Fals-Borda and Rodrigues Brandão, 1986). PAR shares affinities with Action Anthropology, developed earlier by Sol Tax in dialogue with his students (Gee, 2017); both were influenced by Latin American political engagement with research (Rubinstein, 2018) and their aim is to propose a combination of intellectual and popular knowledge that emphasise reciprocity, co-learning, and ethical commitment to community-defined goals. However, PAR emerged within anti-colonial struggles in the Global South and was deeply shaped by Paulo Freire's pedagogy of liberation (Freire, 2014). This implies a dialogical relationship where knowledge becomes a collaborative act of reflection and action (*praxis*), but also a means for human emancipation (Freire, 2014). Therefore, a central point of the PAR methodology is to address structural power struggles and epistemic injustice and to produce a science rooted in social transformation in support of Indigenous and social movements (Bonilla et al., 1972; Rahman, 1983; Fals-Borda and Rahman, 1991; Díaz-Arévalo, 2022). During my fieldwork, my primary aim was to understand how the Guardia Indígena functioned and managed to employ non-violent strategies amidst an armed conflict.

I accompanied the indigenous guards for over a year using a PAR approach, participating in 14 Guardia Indígena actions to manage conflict, collaborating in workshops organised by ACIN in 13 communities, and attending 31 ACIN meetings where immediate political and violent events related to the organisation were discussed. I interviewed 31 indigenous guards and leaders, in addition to informal conversations. Field notes from these events were recorded in writing and included the date, activity, people involved, as well as my own feelings and emotions. With participants' permission, interviews were audio-recorded, transcribed verbatim, and then translated from Spanish to English. When allowed, meetings were also video-recorded and transcribed for analysis. Consent was obtained through the organisation and, in the case of interviews, directly from the interviewees in oral form, as signing a consent form was considered unusual and generated mistrust among participants. After completing my fieldwork, I maintained my relationships with some Nasa leaders and guards and kept up with the news in the region. I have also visited them a few times and joined them in manifestations in Cali, Colombia. Some of the guards I worked with are already dead or have been affected strongly by the conflict. In this article, I aim to honour their hard work.

In the following section, I will describe key practices from indigenous peoples and explain how these practices relate to the concept of *sentipensar* and how this affects their decision-making processes. Moreover, I mention the challenges and learnings I confronted while observing/experiencing these practices and trying to understand them. My experience and, moreover, my fears, were the catalyst that forced me to seek a different approach to my data collection and to begin using the concept of *sentipensar*. As I learned more from their practices and how decisions were made based on a *sentipensar* philosophy.

## Sentipensar in Nasa indigenous practices

### Embodied and collective practices for territorial control

Walking the territory is an ancestral practice and one of the most emblematic activities of the Indigenous Guard. The concept of territory or land, called *kiwe* in Nasa Yuwe (language of the Nasa), is one of the keystones of Nasa indigenous knowledge system. In the Nasa world, the *kiwe* is alive, includes all the beings in it and natural beings, such as mountains, trees, animals, and rivers are powerful. Nasa knowledge is produced and rooted in territory; thus, walking is a way of communicating with the *kiwe*, a way to acknowledge people's connection with it, and a way to learn about themselves. Therefore, for the Nasa people, being knowledgeable and being part of the territory are one and the same.

Indigenous Guards patrol the land regularly to monitor, gather information, and identify potential risks or actors that may endanger Indigenous communities. Through walking, observing, and patrolling, they develop an intimate knowledge of the territory—its pathways, water sources, forests, and wildlife. This is an embodied understanding of the territory: a form of knowledge not acquired through abstract reasoning, but through physical presence, repetition, and relational attention to signs and signals in the environment. This deep territorial familiarity enables them to respond quickly in emergencies, mobilise large groups efficiently, and carry out territorial control activities with greater precision and effectiveness. Territorial control activities are wide-ranging and deeply rooted in the defence of life and territory. They include closing cocaine-producing labs and illegal mining operations, expelling armed groups from Indigenous territories, and searching for missing persons. The Guard also supports community councils, organises security and protection during mobilisations and meetings, and safeguards sacred sites. In addition, they maintain constant vigilance—alerting the community to the risks of bombings or combat—and guard checkpoints at the entrances and exits of the indigenous territories, called *resguardos*.

Most of these activities carry a high level of risk, and guards are unarmed. During my fieldwork, I accompanied them on several operations, including checking illegal mining sites, monitoring the contamination of water sources, trials for community members, or even a trial for FARC members. On the 5th of November of 2014, guard members managed to capture seven heavily armed guerrilla members who had run away through the mountains after killing two guards (BBC, 2014).^1^

That morning, I arrived at ACIN at 10.30 am, and the guards had left earlier to remove some banners and other publicity that the FARC had spread in some indigenous territories. In ACIN, we were receiving information from different guards that a situation had gone wrong and some guards were killed. The phones were ringing. All community members were summoned to join the guards pursuing the guerrillas. “*We are all Guards,”* a leader said to people in the ACIN headquarters. The information was still not clear; we knew that the guards were removing the street banners that, apparently, in one small village called Sesteadero, close to Toribío, the situation had escalated, and that the rebels had shot at some guards for trying to take the banners down. Throughout the day, I saw many cars leaving the area and many leaders mobilising people and I received pieces of information. By the end, we knew that around 400 guards and community members pursued the guerrilla members for several hours, eventually cornering and capturing them to bring them to trial.

‘*At that moment, there were not many guards because the guards were dispersed over all the territory. A person takes more time to give the information than anything else because, after anyone has given the information, the guards come from everywhere. Thus, while we were coming from behind, in the upper part of the mountain other guards were already waiting for the rebels. Practically, here in the territory, those who commit a crime, who kill someone, do not have a way to leave, because guards are going to be everywhere, in each territory, in each path,'* affirmed the coordinator of guards in Toribío.

This shows how they act effectively in uncertain and rapidly changing circumstances. Nasa people rely on having a well-established network and a functioning infrastructure in place. As soon as an alert is issued, it spreads quickly across communities, thanks to the Guardia Indígena's capacity to facilitate communication and coordination, and their knowledge about the territory. The guards are equipped with radios and training, and they are strategically dispersed across different villages and operate as a decentralised network: one person contacts another, who in turn contacts others, creating a chain of rapid mobilisation. In the case described here, the guards alerted people living in the mountains and managed to corner the rebels. At the same time, they called on community members from other areas, mobilising a large number of people who arrived in waves, one after another. This is a horizontal organisation, not one based on individualised command or a top-down hierarchy. Decisions, risks, and outcomes are carried collectively.

The next day, some of the guards told me their version of the story. According to the interviewees, catching the insurgents proved very tense and difficult. Once cornered, one of the insurgents took a grenade and threatened to detonate it. “*We do not get scared with grenades, with that you kill only five of us, and the others will catch you,”* an indigenous leader exclaimed, according to the guards who excitedly told me this. A guard from Cerro Tijeras continued:

‘*At the beginning, I was scared, so I threw myself back when I saw the grenade. I was a little, just a little scared (laughs). First, we were just eight people or nine around the guerrillas, but when the others arrived we started to “get happy”. There were also the brother and friends of one of the killed guards and I saw them very sure of what they were doing... There was also a “mayora” [old lady] who is a guard and looked angry and fearless; she was at the front and seeing her also motivated me. I didn't want to die, but it is like I rub off and “se me sale el indio!” (laughs). Cause, when we are together, we feel more indigenous'* explained the guard.

In this quotation, many indications show the power of collective action and *sentipensar*. One of the guards described how their fear transformed into determination and excitement. The phrase “*se me sale el indio”* spoken with laughter and affect, in this context, encapsulates the embodied and collective dimensions of his Indigenous identity. It suggests that his indigeneity was activated through shared action, emotion, and memory. In the heat of danger, the guard draws strength not from hierarchy or command, but from witnessing the courage of a “mayora,” the grief of kin, and the support of others. Such events are not isolated; they accumulate meaning as part of a longer history of struggle for territory, autonomy, and life.

This moment reflects sentipensar in motion: the blending of feeling, thinking, and acting together, where identity and knowledge are emergent, relational, and embodied. This example shows that *sentipensar* in emergency moments is also about excluding some feelings, and one of the feelings that is not welcome is fear. Indeed, this was very clear in the Guardia Indígena. I observed guards finding fear contagious, like a virus, and to protect themselves from it, they excluded the person who showed fear. After all, being brave is one of the main features of being a Guard and allows them to manage very uncertain situations.^2^ In these situations, the body becomes a protagonist—almost by necessity—as it is exposed to daily risks. As a researcher doing ethnography, I had to take the same risks they were taking. In these situations, the capacity of Nasa people to navigate uncertainty and complexity becomes vital. This is also where *sentipensar* becomes deeply relevant.

### Rituals, señas, and the body as territory of knowledge

Before any mobilisation or territorial control activity, Nasa people and guards must always ask permission from the nature spirits to open the path (*abrir camino*). *Abrir el Camino* is a common practice that involves seeking permission from the nature spirits to undertake specific activities. They look for signs, such as the sky and clouds clearing, as an indication that the spirits have granted permission. If the spirit grants permission, they can proceed. If they find themselves in an emergency and lack time for a proper ritual, they will ensure they get some *chirrincho* (traditional alcoholic drink), offer some to the spirits, and drink some themselves before the walk begins. While walking, many chew coca to sharpen their senses and protect themselves; the guards look for changes or traces in the vegetation and clues that indicate recent events in the landscape. With this information, they should be able to track people or anticipate potential problems and take appropriate measures. The protective power of the coca is well known, as one key informant from Cerro Tijeras casually explained to me when talking about a guard coordinator from resguardo Delicias who had survived at least three assassination attempts. “*He has not been killed because he chews more coca than a cow.”*

There are two main regular rituals practised often among guards: *refrescamientos* (refreshments) and *limpiezas* (cleansing). I interpret *refrescamientos* as rituals designed to protect people from “bad” energy emitted by others, whilst providing them with strength and bravery. Similarly, I understand *limpiezas* as practices that cleanse individuals of their past behaviours, fears, and any burdens they may carry. Both rituals include activities like chewing coca, drinking some traditional alcohol, smoking unfiltered cigarettes, and bathing the body with medicinal herbs. These rituals are performed in specific locations, primarily places with historical significance. They explained to me that this is because those places have a special energy. Chewing coca is another essential part of the rituals, helping the *Thë'Walas* (shamans) to communicate with the spirits. Rituals are performed more frequently when violence escalates or before guards engage in dangerous activities. Thus, ideally, the first thing to do during a dangerous situation is to perform a protective ritual and consult the *Thë'Walas* and the elders.

For the Nasa, it is not appropriate to talk about rituals, they are not meant to be explained but learned through attentive observation. As one leader from Tacueyó explained: “*A shaman never explains to you. The only thing they do is practice, and when you are doing all the rituals you have to pay close attention and learn to conceptualise what the elder tells you.”* Words hold little importance in this context; instead, feelings experienced through the body, called *señas*, are more significant. A *seña* is like an itch that you can feel in a specific part of your body and sometimes it moves. Depending on the way the *seña* moves, *Thë'Walas* interpret them and makes suggestions accordingly. Thus, rituals are not conceptualised through words or abstract reasoning, but through embodied participation—a process of engaging in dialogue with the spiritual world through practice. This embodied, affective, and communal way of knowing exemplifies *sentipensar*: a mode of understanding where thinking and feeling are inseparable. Each participant interprets the ritual based on their own sensations and lived experiences;^3^ yet the ritual itself is a collective practice, rooted in history and guided by spiritual leaders within specific communal contexts. The rituals are an important part of being a guard. Guards expressed it in sentences like: “*...We have to be clean to avoid bad things happening”* or “*I am dirty and, because of that, bad things happened...”* Many told me that the reason the conflict became so violent was that for a while they had stopped performing rituals and begun to forget about their beliefs. These affirmations occur in ordinary conversations and demonstrate that, indeed, for the Nasa, forgetting their beliefs means danger to their communities. Indeed, before the GI was formalised, rituals were almost exclusively for male leaders and elders. Since the guard formalisation, there has been a revitalisation of rituals and beliefs, as now young guards can participate.

Practices such as walking the land, *abrir camino*, chewing coca, and the *refrescamientos* and *limpiezas*, and the meanings attached to these practices in relation to the territory—together with the organisational structure of the Guardia Indígena, show that their ability to act without weapons, even in the face of constant danger, is not simply a matter of strategy or rational planning, as in a military operation. Instead, it is grounded in an epistemology and ethics in which feeling, spirituality, and relational accountability are integral to what counts as knowledge and what legitimises action. This reflects a way of *sentipensar* the territory, where knowledge arises from a deep, embodied relationship with the land, and actions are rooted in collective feeling, spiritual guidance, and shared responsibility.

When I joined the guards and leaders in situations that were considered dangerous, I often felt I was not well-equipped for these activities. I saw them talking about their *señas* in those moments and drinking *chirrincho*. The *señas* are always present and people discuss them. “*I feel a seña,”* they say, identifying where it sits within their bodies and describing it. For example, people say: “*I feel something in my left knee, and it is going up”* or “*I feel something in my ankle, and it is going down.” One* day a female leader told me, “*This morning, my face was itching, and I knew something would make me angry.”* She then recounted having an argument with someone that day, after which she recalled the *seña*. The position of the *seña* within the body and its direction indicate whether it is a positive or negative event coming. Hence, sometimes the *señas* can serve as indicators of future events. The concept of *señas* helped me to understand an idea explained to me later by a female indigenous intellectual: the body is the first territory of knowledge for the Nasa people.

‘*In the Nasa belief system, the body is understood as the first territory, the first structure, and as such, it is sacred, integral, capable of communication, and in need of care. The body communicates through vibrations, pulsations, or “señas”. Señas are messages received in our daily lives—some directed towards the person experiencing them, others meant for different people. The meaning of the message varies depending on the side of the body where it is felt: if it is on the right side, it is positive; if on the left, it is negative. The message also changes depending on whether the seña rises or descends. These messages serve as guides for the self-care of the individual body, the communal body, and the organisational body. For this reason, ritual practice among the Nasa people is closely tied to the feeling of the señas, which allow us to harmonise, balance, cleanse, strengthen, and continue along the path. Through the señas, we can understand what our body is asking of us—often urgently—such as to stop, breathe, and return to the root… In the Nasa worldview, speaking about the body means speaking about wholeness, which challenges perspectives that separate the body from territory and nature.'* Rosalba Velasco, feminist Nasa leader and researcher.^4^

During my fieldwork, I began to pay close attention to my body and see if I could feel a *seña*, but soon I realised it was not beneficial for me, as I became paranoid with the idea of bad things happening. However, as each day was marked by uncertainty, and circumstances could quickly shift from calm to violent, I developed a habit of scanning my body each morning to assess how I felt—emotionally and physically. I would try to locate discomfort or tension and reflect on what it might mean. It was as if I were learning to interpret my own *señas*, tuning into the signals my body offered. For me, the *señas* became cues from my body that told me about the environment and how I felt in that precise context. When I felt strong and confident—my body light and open—I would join the guards in participatory observation. On days when I felt heavy, anxious, or fatigued, I stayed at the ACIN headquarters, focusing on my role as a communication supporter. Looking back, I now understand that this practice of bodily attunement was the first time I consciously began to use a *sentipensar* approach to guide my fieldwork. It became an essential tool for navigating a violent environment and for connecting with the Nasa people in ways that acknowledged both our differences and resonances. Moreover, I could not abandon my academic framework, rather, I felt I was navigating between two epistemic worlds that remained distinct but co-present. I felt like a *mestiza* encountering what I would later come to know as *ch'ixi* epistemology. At the time, I lacked the vocabulary to articulate this, but I was already feeling its weight in my body and my choices.

### Assemblies and the politics of sentipensar

During fieldwork, negotiations for the Peace Agreement between the Colombian Government, represented by President Juan Manuel Santos, and the leftist guerrilla group FARC-EP took place in Havana, Cuba, while guerrilla members continued to fight in many regions, including northern Cauca. The violence and uncertainty were exceptionally high, representing one of the main characteristics of this region. During this period, meetings were held frequently among ACIN, community members, and among indigenous organisations in general. Meetings known as Context Analysis usually last for 1 day or several hours and serve to share urgent information. These meetings are open to the public and are often quite crowded, with journalists and researchers participating in relatively passive roles. They are intense gatherings, filled with information, analysis, disagreements, and discussions.

General Assemblies—whether at the *resguardo*^5^ or organisational levels (such as CRIC or ACIN)—are central to the political life of the Nasa people. These gatherings, often extend over several days and exemplify what Mouffe (2006) call as radical democracy: a space where decisions emerge from extended dialogue, embodied presence, and the negotiation of diverse voices and tensions. One constant feature in these assemblies is the *Tulpa*^6^, a fire built with three stones. The *tulpa* is not always placed at the physical centre of the gathering, but holds a quiet, persistent centrality in practice. People come and go from the fire, there they chew coca, use medicinal herbs, sit in silence, or talk with the *Thë'Walas* who often guide the gathering. The *tulpa* is an ancestral practice that was once central to every household, as one key informant explained when recalling her childhood:

‘*My grandparents, in those times, they had their tulpa. They made their appeals to the spirits, they made the offering to the spirits. But they did not explain that to me. I understand today and begin to remember what they were doing... As I experienced it when I was a child, for me it is very easy to understand. For my daughters, it is difficult, as I do not practice it because, unfortunately, everything has changed a lot. Sometimes when we talk, we make a chocolatada*^7^ *for them to learn. While with my parents we chewed coca and they called and offered it to the spirits. If you see? I lived it, so today I understand it much more. Now, to my daughters I have to explain it and do a little practice, doing the things that can be done.'*

During a major CRIC assembly, I asked an elder sitting by the tulpa about its meaning. He explained:

‘*Our parents and ancestors, without having much technology, without knowing about the city or the white people, had three tulpas in their house. They would place the small pot there to cook meals, and the mother, father, and children would sit around it, and everyone would talk there, and there was no interference. We Indigenous communities had this sacred practice. Even when doing work, holding a minga*^8^, *we did the same. This comes from our ancestors, and the sacred fire is lit not with matches or candles, but with stones or by rubbing the chonta tree on a yellow guayacan.'*

These quotations show that the Tulpa served not only as a place for cooking but also as a space for storytelling, reflection, and family connection, anchoring daily life in spiritual and communal meaning. Nowadays, the Tulpa serves an important part of Assemblies. The fire becomes a place to pause, to reflect, to reconnect—both spiritually and emotionally. In this sense, the *Tulpa* is not only symbolic but functional: a space to *sentipensar*, to weave together thought, feeling, and collective presence. However, many of these practices are at risk. As mentioned in previous quotations, the *tulpa* was once a central feature in every household. During a major CRIC assembly, I asked another elder sitting by the *tulpa* why it was important. She explained:

‘*If, for example, there's no tulpa held by an elder at an Indigenous event, it's a sign that our people are already being lost—that ancestral knowledge is running out. There would no longer be anyone to speak for those who remain, no one to teach “lo propio” (our own ways), no one to explain… why it's thundering, why there are strong winds, why there's wind with rain, why the rainbow appears. And then, ancestral communication would be lost. That's why people come to the tulpa, ask for medicine, sit to listen to what we're talking about… they chew the sacred coca leaf, and in this way, people come to understand what it means to respect Mother Nature.'*

As this elder explained, the tulpa is not simply a place for rest or ritual—it is a critical epistemic site to share “lo propio.” If it disappears, “ancestral communication would be lost.” The tulpa, then, becomes a space of intergenerational learning, where young people listen, ask for medicine, and learn to “respect Mother Nature” through the embodied practice of chewing coca and sharing stories. This relational and sensory mode of knowledge transmission reflects a form of *sentipensar*—a way of knowing that cannot be disentangled from presence, practice, and spiritual connection.

The *Tulpa* plays a key role in the political practices of the Nasa people. Community meetings and assemblies are not purely procedural or discursive events. The tulpa shows that they are spaces where knowledge emerges from a *thinking-feeling* relation to territory, history, and communal life. In this way, *sentipensar*, most often associated with embodied and spiritual ways of knowing, also informs the political and organisational practices of the Nasa people—particularly in the collective spaces where decisions are made.

These gatherings often begin with rituals, invoking the spirit of the land and the guidance of ancestors and the spirits. Elders and spiritual authorities speak not only from experience but from embodied wisdom and visions. Youth and guards contribute through what they observe and sense while walking the territory. Deliberation, in this sense, is not abstract debate but an expression of *sentipensar*—a collective act of discerning what needs to be done by listening with the body, with ancestral memory, and with the land. This is why *sentipensar* should be understood not only as an epistemology of inner or ritual experience, but also as a political epistemology: a way of collectively thinking-feeling-acting that underpins Indigenous governance.

## Sentipensar as an epistemology

Previous research on the Nasa people has primarily examined how Indigenous communities mobilise to reclaim their rights to land, resources, and self-determination, often in resistance to colonial and neo-colonial power structures (Findji, 1991; Rappaport, 2002; Hristov, 2005, 2009; Chaves et al., 2020). A common thread in this body of work is the centrality of land—or *territory*—as a core component of Nasa identity and struggle. Massey's (1995) presents a relational view of space, which sees identity and social relations as produced through geographical practices, and how these processes can be strategically employed to foster more participatory and equitable forms of democracy. Drawing on this definition, I understand that it is not the territory but the practices that produce the political effect, and in this case it is the practice of *sentipensar* that is used for decision-making. However, it is important to acknowledge that political process that embraces *sentipensar* requires time and energy. The priority lies not only in effectiveness or achieving goals but also in the process itself. This process is not only based on information and cost-benefit analysis but also encompasses the embodied experiences of community members, the *señas* in the body, and the signs from the spirits. Information, therefore, is rich, taking into account not only facts but also expressions that arise from diverse sources. There is nothing mechanical about these practices in the Nasa indigenous people, which is why the concept of *sentipensar* is particularly relevant. It involves being present in the moment, and genuine presence includes feelings in the body, a connection with the moment and space, and links with others while thinking.

From a Western perspective, we might downplay Nasa practices, interpreting them as socio-psychological tools to deal with uncertainty or, as Damasio (1999) suggests, from a neurological point of view, we could argue that emotion probably assists reasoning, especially when it comes to personal and social matters involving risk and conflict. This author goes further and suggests that the term “feeling” should be reserved for the private, mental experience of an emotion, while the term “emotion” should be used to designate the collection of responses, many of which are publicly observable (Damasio, 1999, p. 295). This position reinforces a Western separation of public and private realms, or yet another dualism. Moreover, it shows the limitation of ontologies based on individualism. Nasa practices go beyond this idea of emotion assisting individual reasoning, showing that feelings are essential processes that help individuals reconnect and restore balance with their communities. It is about restore connections. Translating *sentipensar* into a Western concept is not simply about misinterpretation; it's about epistemic injustice: the inability of dominant conceptual frameworks to even recognise the full meaning of other ways of knowing.

In this way, this research contributes to ongoing discussions in decolonial theory, Indigenous studies, and epistemic justice by showing how the concept of *sentipensar* is not only an alternative to dominant academic paradigms but a living epistemology embedded in the practices of the Nasa people. The three sets of practices presented—territorial control, rituals and body knowledge, and collective assemblies—are interconnected expressions of an alternative epistemology: one that challenges the binary logics of Western modernity. A way of knowing that is deeply embodied, emotionally attuned, and communally anchored. The concept of the body as the first territory among the Nasa, as explained by the Nasa leader, collapses the division between the physical and the epistemic. In territorial control activities, rituals, and general assemblies knowledge is generated through walking, feeling, sensing—through a body that is very active and present and never isolated but in relation to land, history, and others. *Sentipensar* is then an example of a relational, embodied epistemology deeply rooted in territory, and community. Moreover, the *tulpa* in the general assemblies reveals that *sentipensar* is not just personal or ritual—it is also political. Deliberation is not limited to argumentation or logic or cost benefit analysis; it includes listening with the body and consulting the spirits. This identifies indigenous Nasa governance not as procedural neutrality but as an ethical, affective, and epistemic engagement with the world. Moreover, these practices reflect what Escobar (2018a) calls ontologies of radical relationality and interdependence, where the human is always in relation to territory, ancestors, non-human beings, and the collective.

These results can be linked to many academic discussions in different fields (Mouffe and Holdengraber, 1989; Massey, 1995; Nussbaum, 2004; Paechter, 2006; García Selgas, 2014; Escobar, 2018a; Bastidas Aguilar, 2020), but I will focus on two that are particularly important for researchers doing fieldwork with indigenous people. First, a decolonial approach addresses oppression as part of deep and historically rooted ontological conflicts that started with colonisation (Mignolo and Escobar, 2010). In the introduction, I questioned epistemic dualisms of the rationalism and the Cartesian tradition (Escobar, 2018a) because these epistemic dualisms have been imposed in most academic institutions as the standard. Therefore, many intellectuals acknowledge the ontological conflicts, for instance, between Western researchers and indigenous people (Blaser, 2013). Ontological conflicts emphasise that some conflicts are not about people's beliefs but about different realities being done in different practices (Law, 2015). This perspective is analytically radical because it treats what is considered “real” as the effect of contingent and heterogeneous enactments, performances, or relational configurations (Law, 2015). In this view, foundational categories such as “nature” and “culture” may not hold the same meaning—or may not exist at all—in these alternative worlds (Law, 2015). The ontological conflicts occur when actors from distinct ontologies engage with each other, leading one side to overlook or disregard elements that belong to the worldview of the other actors (Escobar, 2016). However, ongoing processes of colonisation and globalisation have not only shaped relations between Indigenous and non-Indigenous worlds, but have also contributed to ontological tensions within Indigenous communities themselves—between generations, geographies, or ways of relating to land and tradition. A case in point is the *tulpa*. Once a central element of daily life in Nasa households, it has become less familiar to younger generations. At the same time, it has gained new significance as a site of collective political deliberation in general assemblies. Rather than disappearing, the *tulpa* has migrated across social spaces—showing that these ontological worlds are not static but adaptive. Moreover, defining ontological conflicts as a phenomenon occurring solely between indigenous peoples and Western or Westernised researchers risks rendering the discussion essentialist. If ontological conflicts highlight the incommensurability of realities that exist in different places and practices, our role as researchers is to find the conceptual tools to move from ontological conflicts to ontological dialogues. The proposal here is to find partial connections—points where communication or understanding is possible, but without assuming a shared ontology (De La Cadena, 2015).

Law (2015) explains that different realities are enacted in practice, even inside Western societies. If we understand scientific research as a contextual *construction of social, material, and discursive networks* of human actors and non-human actors who stabilised, and validated this knowledge through the interactions within these networks (Latour, 1994). Consequently, a decolonial perspective compels us to try to understand the social, material, and discursive networks and processes within the Indigenous ontologies that generate knowledge. This implies treating Indigenous knowledge systems with the same analytic respect as modern science, and not merely as cultural artefacts or belief systems. Here, the collaboration between indigenous researchers and intellectuals with Western or Westernised researchers is crucial (Rappaport, 2004, 2005). Implicit in this argument is a commitment to ontological pluralism, in which different networks of knowledge production are not merely culturally distinct, but operate within different realities—e.g., where rivers are ancestors, where knowledge comes through dreams, or where ritual is a method of epistemic stabilisation. This is essential because it represents a means to preserve and enable different ontologies to flourish, avoid paternalistic viewpoints, and promote respectful ontological dialogues.

This brings me to the second point of discussion, the importance of positionality for acknowledging our limitations, the origins of our interpretations, and the particular opportunities and constraints we bring to the research process. By explaining our positionality, we reflect an ethical concern with the potential misinterpretations we might make when engaging with data, and it promotes transparency in how knowledge is produced. Furthermore, we make it possible to trace how our experiences in the field are connected to our own ontological and epistemological foundations. This also allows the reader to critically reflect on their own interpretations in relation to ours, fostering a dialogical and reflexive reading of the research. In this article, I propose considering the issue of *mestizaje* as a case of internal ontological conflict. This is not a wish to deny the incommensurability of ontologies, but to recognise *mestizaje* as a type of ontological and methodological bridge (Anzaldúa, 1987; Lugones, 1992; Smith, 2005, 2008; Rivera Cusicanqui, 2018). Three authors have influenced my perspective as a *mestiza*, Anzaldúa (1987) theorised *mestiza consciousness* as a form of knowledge grounded in ambiguity, pain, and emotion, where the body becomes a site of insight; Rivera Cusicanqui (2018) with the *ch'ixi epistemology*, and Sara Ahmed (Ahmed and Stacey, 2001; Ahmed, 2014) on how emotions circulate and shape orientations, producing collective forms of knowledge and political belonging. These conceptual perspectives framed my positionality and guided my understanding of the concept of *sentipensar* in Nasa peoples' practices. I approach this work through a *ch'ixi* epistemology, which allows for the coexistence of multiple, even contradictory, ways of knowing without forcing them into synthesis. This framework is particularly important when engaging Indigenous ontologies, which must not be romanticised or essentialised. A *ch'ixi* perspective makes it possible to affirm the relational, spiritual, and territorial dimensions of Indigenous knowledge, while also holding space for internal critique—such as the operation of gendered hierarchies or the reproduction of colonial logics within Indigenous contexts themselves. It is here that decolonial feminism and standpoint theory become vital, offering analytical tools to navigate these tensions without falling into dualistic or universalist traps (Anzaldúa, 1987; Lugones, 1992, 2024, 2010; Smith, 2005, 2008; Méndez Torres et al., 2013; Kiran, 2017; Rivera Cusicanqui, 2018, 2020). Here, the point is that instead of “studying” indigenous people, Western researchers work together with indigenous intellectuals to create ontological dialogues and co-creating knowledge in ways that respect complexity, contradiction, and situated accountability.

## Conclusions

While critical Western traditions have offered important insights into embodiment, emotion, and relationality in knowledge-making, the concept of *sentipensar* brings a unique contribution by integrating these dimensions into a single, lived epistemic mode. Rooted in Indigenous and Afro-descendant worldviews in Latin America, *sentipensar* emerges not as a theoretical construct but as a practical, collective, and ritualised form of knowing. The practices from the Nasa people show that the concept's strength lies not only in what it critiques—namely, the fragmentation and abstraction of dominant epistemologies—but also in what it affirms: the relational, affective, and embodied processes through which communities generate meaning in the face of uncertainty, violence, and colonial legacies. In this way, *Sentipensar* for the Nasa is an epistemology.

Nasa practices show that their knowledge can emerge from being within relationships through the body, through affect, through intuition, and through deliberation processes that listen the body and the spirits, a memory carried collectively across generations. It affirms that knowledge is not only produced in the mind, but also in rituals, in land-based practices, and in the everyday acts of care and resistance that sustain community life. As such, *sentipensar* offers an understanding of knowledge that is deeply situated and communal, challenging the dominant view of knowledge as a cognitive or individual pursuit.

In this way, *sentipensar* stands as a liberatory epistemic tool that challenges the fragmentation characteristic of Eurocentric scientific rationalism. Far from being a metaphor, *sentipensar* expresses an ontological orientation towards the pluriverse, a world in which multiple ways of being and knowing coexist without hierarchy (Escobar, 2018a). It is not merely an individual way of knowing but a collective, situated, and ancestral epistemic practice. As such, it offers not only a method for engaging with communities, but also a powerful decolonial critique of dominant knowledge systems, inviting researchers to feel-think with humility, relational accountability, and care. In this article, I also have reflected on how, through living and working with the Nasa people, I came to engage with the concept of *sentipensar* not as a theoretical abstraction, but as a lived and relational way of knowing that reshaped my approach to fieldwork. It became an epistemological approach. As a mestiza using a *ch'ixi* epistemology my role is to work together with indigenous intellectuals to create ontological dialogues.

Importantly, *sentipensar* does not emerge from academic critique but from traditions of resistance, healing, and survival under colonial and neo-colonial conditions. Its value lies not only in what it reveals—uncertainty, contradiction, and relationality—but in how it is practised: through walking, rituals, storytelling, and protective actions that sustain community meaning-making in the face of systemic violence. As such, *sentipensar* represents a decolonial epistemology in its own right, offering alternative ways of producing, transmitting, and embodying knowledge.

## Final reflection

In a time when artificial intelligence increasingly shapes knowledge production, decision-making, and academic research itself, *sentipensar* reintroduces what these systems often suppress: the deeply human capacity to know through feeling, connection, and situated reflection. Nasa practices remind us that in contexts of profound uncertainty, risk, and conflict, rational calculation is not sufficient; relational wisdom, affective attunement, and community-based judgment become indispensable. This invites us to recognise the limits of technoscientific reason. As such, Nasa practices challenge us to reimagine what it means to know, to relate, and to be accountable in a *pluriversal* world. This is the ethical and political task that *sentipensar*, as a decolonial and relational epistemology, makes visible.

## Data Availability

The raw data supporting the conclusions of this article will be made available by the authors, without undue reservation.

## Footnotes

^1^For a complete description of the events, please check my previous article about how the Guadia Indígena organise for emergencies (Chaves et al., 2020).

^2^For a detailed research on how the guards construct a warrior identity please check (Chaves et al., 2019).

^3^While writing these words, I realised I was missing the point myself. To honour the practice I was describing, I paused and performed a personal ritual. I felt I needed permission from the Nasa people. I lit incense, turned off the lights, and transported myself back to the rituals I had once participated in. The final description is drawn from field notes now enriched by the embodied moment in which I write: by the smell of the incense that brought me back to Cauca, and by the memory that returned through the body before it reached the page.

^4^This excerpt is taken from the abstract: *Integrando Conocimientos: El Cuerpo Desde El Universo Nasa* presented by Rosalba Velasco in the LASA 2025 conference.

^5^The resguardos are indigenous territories recognised by Colombian government as communal, unavailable for sale or rent, and governed by indigenous authorities or *cabildos*.

^6^Tulpas are the three hearthstones in a traditional Nasa wood fire. Their name derives from the tul, which refers to the principles that govern the community, such as solidarity, conservation, autonomy, resistance, respectful use of nature, harmony, spirituality, reciprocity, and good management (Yule, 2012).

^7^A chocolatada is a meeting to drink hot chocolate together.

^8^A *minga* is a traditional form of communal work rooted in reciprocity, where labour and food are shared among community members. While historically focused on tasks like farming or construction, in contemporary Colombia, Mingas also serve as political gatherings, protests and spaces for collective decision-making.
